# Debonding Detection in Grouted Sleeves Using Axisymmetric Longitudinal Guided Waves

**DOI:** 10.3390/s23229134

**Published:** 2023-11-12

**Authors:** Jiahe Liu, Dongsheng Li, Xiushi Cui

**Affiliations:** 1 School of Civil Engineering, Dalian University of Technology, Dalian 116024, China; 1104060917@mail.dlut.edu.cn (J.L.); jazzycui@mail.dlut.edu.cn (X.C.); 2 State Key Laboratory of Coastal and Offshore Engineering, Dalian University of Technology, Dalian 116024, China

**Keywords:** guided waves, numerical simulation, grouted sleeve, debonding detection, arrival time

## Abstract

Grouted sleeves (GSs) are a type of precast joint that can effectively connect steel rebars with excellent performance. However, the grouting debonding problem, which can occur due to the leakage of the glue plug, can seriously affect the properties of GSs. In this paper, a guided-wave-based structural health monitoring (SHM) method is used to detect debonding in GSs. The axisymmetric longitudinal mode is selected as the incident wave since it is sensitive to axial damage. Eight piezoelectrics (PZTs) are then symmetrically installed to actuate signals. The proposed samples are GSs with four different debonding sizes. First, the relationship between the arrival time of the first wave packet and the debonding size is explored through theoretical derivation. The arrival time decreases linearly with an increasing debonding size. A similar trend is observed when the relationship is verified via a numerical simulation and experimental results. This method will provide a reference for detecting debonding in similar GS multilayer structures.

## 1. Introduction

Prefabricated buildings (Pbs) have numerous advantages, such as being low in cost, energy efficient, less environmentally impactful, and quick to construct [1]. The connection of the reinforcement of adjacent prefabricated elements is a vital part of Pbs to ensure integrity. GSs are the most widely used in Pbs for connecting prefabricated elements due to their strength and performance [2,3,4,5]. For example, GSs are often applied in prefabricated elements, including shear walls [2], frame columns [3], and frame beams [4,5], and they can achieve equivalent mechanical performance as cast-in-place structures while having more advantages in construction. The core of GS technology is transferring stress among prefabricated elements by filling them with high-strength grout; however, grout leakage triggered by poor adhesive bonded plugs or a hostile environment usually occurs near the steel sleeve [6,7,8].

Grout leakage can greatly increase the probability of debonding between a steel sleeve and grout, which reduces the bearing capacity of a GS. For example, Liu et al. [9] determined that the failure of the confinement effect of the steel sleeve on the grout is the direct cause of the shear transfer failure. Therefore, long-term and reliable detection is needed to ensure that GSs do not undergo debonding. The detection of grout debonding is difficult because the grout in GSs is covered by steel sleeves [10,11]. Thus, intuitive visual detection and most traditional non-destructive testing methods cannot be applied directly.

The authors of this study reviewed the detection techniques of GS defects in their previous work [12] and found that most of the current detection techniques are only used to detect the presence of defects, not to quantify them. Li et al. [13] and Ma et al. [14] found that using 150 kHz ultrasound can accurately distinguish between grouted and ungrouted GSs. Yao et al. [15] evaluated the quality of grouting in GSs via the energy received by a damping sensor, and they considered the engineering quality to be substandard if the energy was higher than 180. In addition, [12] indicated that the guided wave technique has more advantages than traditional ultrasound methods in detecting longitudinal debonding in GSs because of the longer propagation distance and lower attenuation [16].

The time lag between the two wave packets in a guided wave, i.e., the time-of-flight (ToF), is commonly used as an indicator when a guided wave detects internal debonding in structures, such as between steel rebar and concrete, a steel plate and concrete, and a steel plate and fiber cloth [17,18,19]. Among them, Zima et al. [17] used the flexural mode wave to derive the exact relationship between the wave ToF and debonding size. Aseem et al. [18] showed that the ToF of nonlinear guided waves decreased with increasing size of debonding between steel rebar and concrete and demonstrated that the damage location could be determined. The main reason for the ToF changes caused by debonding is related to modal conversion in the wave propagation path [19]. Generally, guided waves travel faster in separated steel sleeves than in GSs. Hence, when grout debonding occurs, a portion of the wave energy propagates along a separated steel sleeve, which implies a decrease in the ToF. 

In this paper, guided waves are implemented on the surface of a GS, and a ToF is used to determine the defect length along the GS. The proposed method enables the detection of debonding defects that have not been addressed by previous researchers, as well as the quantification of the defects. In addition, the method allows for long-distance detection along the sleeve surface, overcoming the shortcomings of traditional ultrasonic methods that only offer point-by-point measurement of sleeve grouting defects. The structure of this paper is as follows: In Section 2, the dispersion curves of the GS are developed, and the working mode of the guided wave is analyzed. In Section 3, the propagation and reflection of longitudinal guided waves within the finite element model of a GS are demonstrated, and an extraction method is introduced. In Section 4, the finite element model in Section 3 is validated using experiments. Finally, in Section 5, this study is summarized, and future prospects are provided.

## 2. Materials and Theoretical Method

### 2.1. Configuration of the GS Models

**Figure 1:**
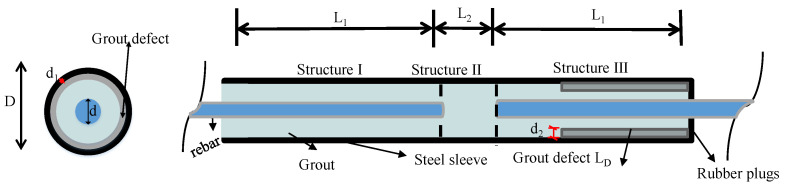
Details of the GS configurations.

In the post-cast band structure, GSs have a complete inspection surface because they are cast with concrete only after the grouting material is completed. For this reason, the concrete protective layer is not considered in this paper, and for generality, the GS size was selected to be a moderate rebar diameter (18 mm), as shown in Figure 1 and Table 1.

In addition, debonding defects are investigated by evenly pasting 2 mm thick polystyrene foams on the inner surface of a GS, with lengths of 0, 72, 144, and 288 mm. The GS size is shown in Table 1. The geometric dimensions and material properties (Table 2) of the GS are provided in detail so that other scholars can directly draw the dispersion curves and better analyze the theory proposed in this paper.

### 2.2. Dispersion

Guided waves are dispersive due to the constrained medium boundary, which means that the group speeds of wave modes depend on the excitation frequency and properties and geometrics of the media [20]. Three types of guided waves exist in GSs: longitudinal, flexural, and torsional modes. Longitudinal waves cause most particles to move axially and longitudinally, while torsional waves mainly cause particles to move in a circular motion [21]. Therefore, longitudinal waves are more sensitive to the debonding along the GS. Moreover, specially designed PZTs can eliminate torsional waves, leaving only longitudinal and flexural waves; however, flexural waves cannot be removed but can be reduced by using multiple PZTs [17,22]. The following dispersion curves will only account for flexural and longitudinal waves.

**Figure 2:**
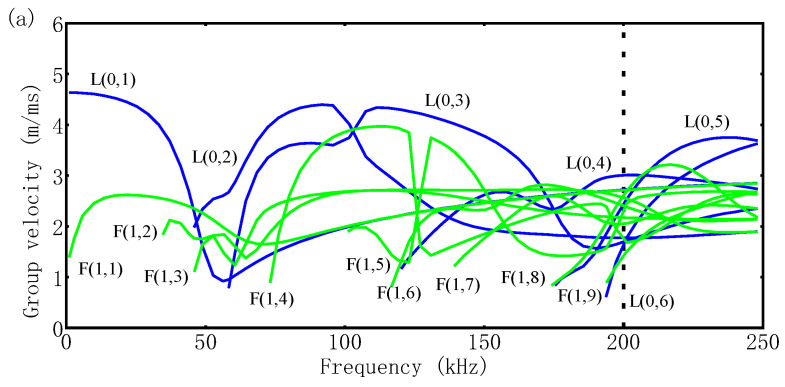

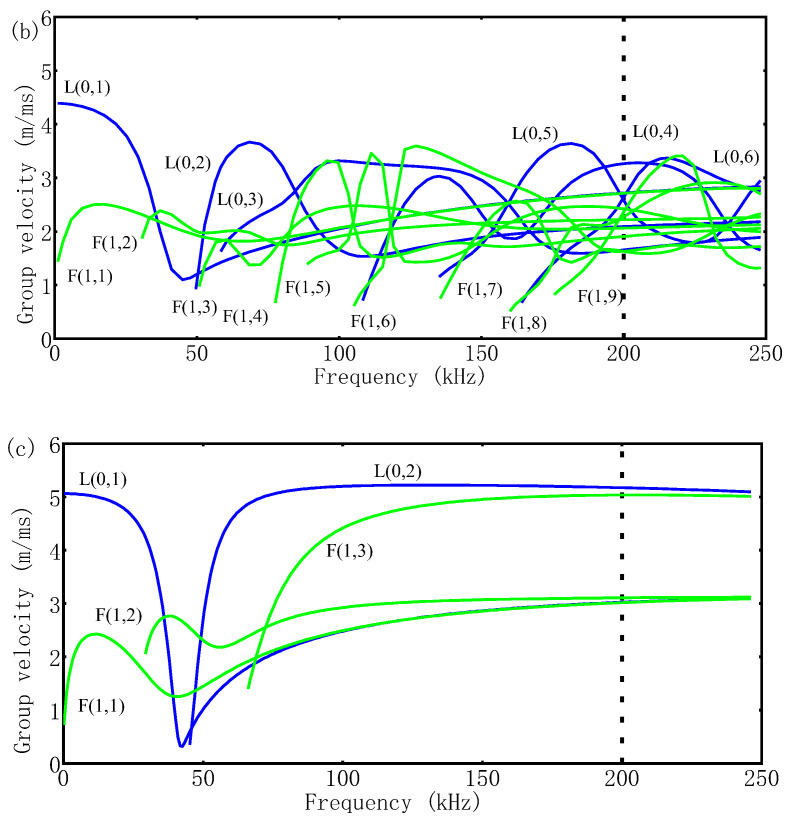
Dispersion curves (group velocity—frequency) for (**a**) Structure I, (**b**) Structure II, and (**c**) Structure III of the GS.

Because the dispersion curves for the three structures of the GS (Figure 1) and, consequently, the group velocities differ, the speeds can be monitored to effectively detect debonding, which is the central premise of the methodology in this paper. Therefore, knowledge regarding dispersion is essential for analyzing wave velocity change-induced defects. Guided wave dispersion curves (i.e., group velocities vs. frequency) can be calculated using an open-source PCDISP [23]. The group velocities for waves propagating in the three structures of the GS are shown in Figure 2. It can be seen that when the GS undergoes debonding, the number of guided wave modes decreases, and the dispersion becomes weaker mainly due to the simpler cross-section of the waveguide propagation structure.

The frequency of the dispersion curve is only calculated up to 250 kHz based on the following two reasons: a higher frequency would cause more modal overlap and bending waves [24,25], and ultrasonic waves would have large attenuation in cementitious materials, as shown in Figure 2, when the ultrasonic wave speed is approximately 3000 m/s, the frequency is 250 kHz, and the wavelength is approximately 12 mm. At this time, the wavelength is more than 10 times the grout particle size. Thus, the ultrasonic wave is in the Rayleigh region, and the dispersion and attenuation phenomena can be effectively controlled [26].

### 2.3. Theoretical ToFs under Different Debonding Scenarios

**Figure 3:**
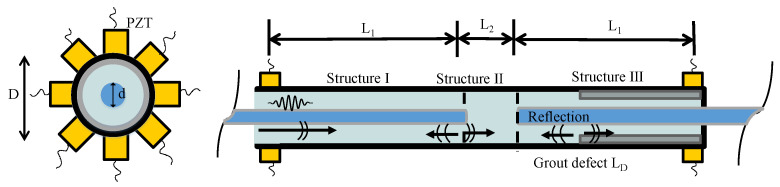
Schematic diagram of the wave propagation in the GS.

Since the faster wave is recorded in the signal first, only the first wave will be considered in this paper. During wave propagation, as depicted in Figure 3, guide waves travel along the GS containing the entire area (Structure I), the middle of the GS (Structure II), and the steel sleeve (Structure III). When the first wave encounters damage, mode conversion will occur. Some of the waves will be reflected, while others will continue to travel along Structure III. The waves in Structure III are separated into two parts that travel separately along the steel sleeve and the bonded GS. Subsequently, only the waves that propagate in the steel sleeve can be sensed because of the location of the PZT receivers. 

The velocity of the waves traveling along the steel sleeve is usually higher than that on the undamaged GS. Based on the description of wave propagations, the corresponding relationship between the ToF of the first wave and the debonding length can be derived as follows:

(1) $$\mathrm{ToF}=\{\begin{array}{cc} \frac{2L_{1}-L_{D}}{v_{1}}+\frac{L_{2}}{v_{2}}+\frac{L_{D}}{v_{3}}=L_{D}(\frac{1}{v_{3}}-\frac{1}{v_{1}})+(\frac{2L_{1}}{v_{1}}+\frac{L_{2}}{v_{2}})\; & L_{D}\geq L_{1}\\ \frac{L_{1}}{v_{1}}+\frac{L_{2}-(L_{D}-L_{1})}{v_{2}}+\frac{L_{D}}{v_{3}}=L_{D}(\frac{1}{v_{3}}-\frac{1}{v_{2}})+(\frac{L_{1}}{v_{1}}+\frac{L_{2}+L_{1}}{v_{2}})\; & L_{1}<L_{D}<L_{2}\\ \frac{L_{1}-(L_{D}-L_{1}-L_{2})}{v_{1}}+\frac{L_{D}}{v_{3}}=L_{D}(\frac{1}{v_{3}}-\frac{1}{v_{1}})+\frac{2L_{1}+L_{2}}{v_{1}}\;\;\;\; & L_{D}\leq L_{2} \end{array}$$

where $L_{1}$ is the distance between the center of the PZTs and the steel rebar boundary. $L_{2}$ is the middle of the GS. $L_{D}$ denotes the ratio of the defect size. $v_{1}\;$is the velocity of the guided waves in Structure I; $v_{2}$ is the velocity of the guided waves in Structure II; and $v_{3}$ is the velocity of the guided waves in Structure III. As shown in Equation (1), since both velocities and distances are known, an approximately linear relationship between the ToF and the defecting length can be obtained. 

The choice of working frequency should take into account three factors: scattering attenuation, the guided wave mode, and resolution. As discussed in Section 2.2, the frequency is chosen to be below 250 kHz to minimize the attenuation due to scattering. Moreover, as illustrated in Figure 2, the bending wave velocity at this frequency is much lower than that of the longitudinal wave, which simplifies the extraction of the Tof. Lastly, the resolution should be considered, which is inversely proportional to the frequency. A low resolution can decrease the mode number and attenuation, but it cannot offer high resolution, which hinders the quantitative characterization of defects in the following step. Hence, in this paper, 200 kHz is adopted as the excitation frequency. The velocity of the first wave presented at 200 kHz is shown in Table 3.

## 3. Numerical Analysis

### 3.1. Numerical Model

To visualize the phenomenon of wave propagation, numerical investigations were performed. Three-dimensional numerical simulations of guided wave propagation in the GS were performed in the ABAQUS software environment. The GS was introduced by tying the grout to the steel sleeve and rebar. To simulate the defect conditions, corresponding parts of the grout near the steel sleeves were deleted, while other areas were maintained with “tie” constraints. Eight-node linear brick elements with dimensions of 1 mm × 1 mm × 1 mm were used. 

**Figure 4:**
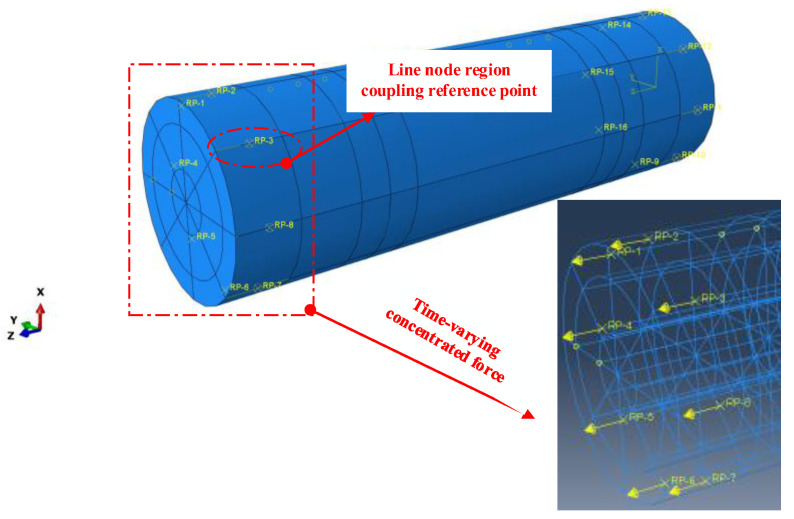
Numerical model of the GS.

Usually, a narrow-band burst can minimize dispersion [27]. Hence, a 200 kHz five-cycle Hanning window tone burst was employed in the numerical study. A fixed time step of 0.01 us was set for all numerical cases. For simplicity, the excitation signal was introduced to the numerical model through the time-dependent concentrated forces on the modes of the actuators, while the input signals were obtained by averaging the stress values of the chosen nodes of the receivers. In addition, the PZTs and the coupling agent did not have separate element types, and the applied force acted directly on the GS, as shown in Figure 4.

### 3.2. Numerical Results

**Figure 5:**
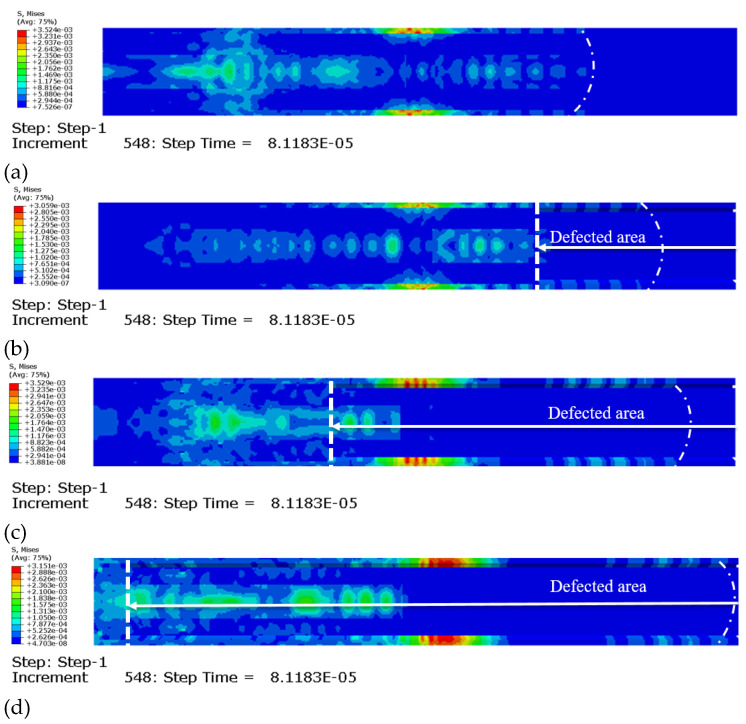
Numerical stress of the wave propagation on the damaged GS (**a**) in the 0D-debonding model, (**b**) in the 6D-debonding model, (**c**) in the 12D-debonding model, and (**d**) in the 18D-debonding model. “8.1183E-05” means “8.1183 × 10^−5^”.

Figure 5 shows the wave field snapshots at the same time for different defect sizes (S_1, S_2, S_3, and S_4). It can be clearly seen that various modes are distributed in the GS. After the first wave encounters the defect area as dotted lines, some waves are reflected, while other waves are divided into two parts and spread forward, which supports the description of the theoretical phenomenon. The wave propagation in the defective samples is faster than that in the healthy samples. It is also apparent that the larger the lengths of the defects, the faster the wave velocities, which coincides with the ToF trend in the theoretical derivation. 

#### Subsubsection ToF Extraction Method

**Figure 6:**
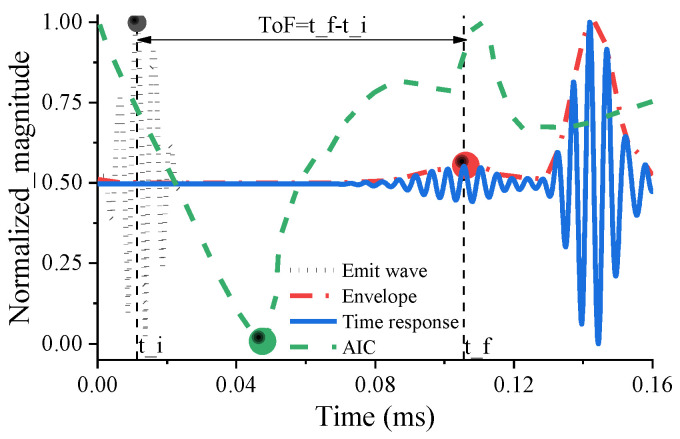
The extraction process of the Tof: the green circle is the AIC starting point of the first wave of the received signal, and the red and black circles are the extreme points of the first wave of the received and transmitted signals, respectively.

The Tof value is obtained by calculating the time difference between the peak of the emitted wave and the first peak of the received wave. After obtaining the ultrasonic signal, the signal is first enveloped to find all the maximum points of the signal; then, the starting point (t) of the ultrasonic signal is roughly obtained with AIC (Akaike’s information criterion). Finally, by comparing the waveforms, the first maximum point exceeding (t) is found as the reference point of the received wave (t_f). The calculation of the TOF involves subtracting the time (t_i) corresponding to the peak of the emitted wave from t_f (ToF= t_f- t_i). The visualization process is shown in Figure 6. 

**Figure 7:**
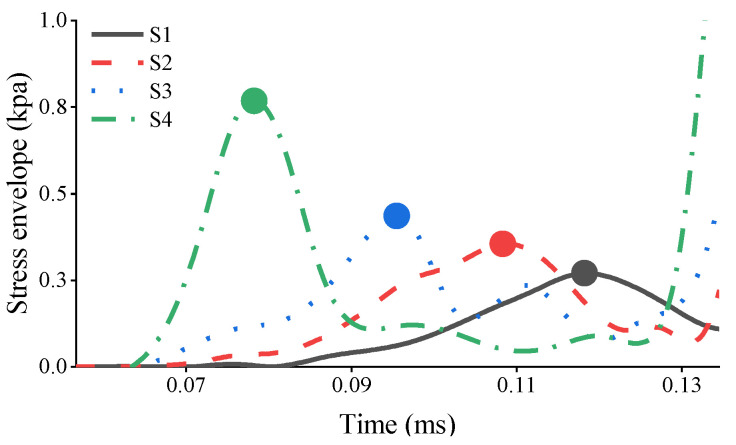
Numerical results of the received signals in the GS.

Figure 7 shows the upper envelope diagrams of the ultrasonic signals of the four models, and the peak points of the first wave are marked by circles. With the increase in the debonding size, the first wave is clearly shifted forward in agreement with the theoretical derivation. Subsequently, the peak points in the first wave packet are extracted to calculate the ToFs, as shown in the circles in Figure 7. The ToFs are displayed in Table 4.

## 4. Experimental Analysis

### 4.1. Experimental Setup

To confirm the theoretical results, experimental studies were subsequently conducted. GS samples with the same geometry and material properties as those prepared in Section 2 were used. Polystyrene foams were first input into the steel sleeve to introduce defects. Afterward, the already mixed grout was inputted into the steel sleeve through an injection gun until the sleeve was filled.

**Figure 8:**
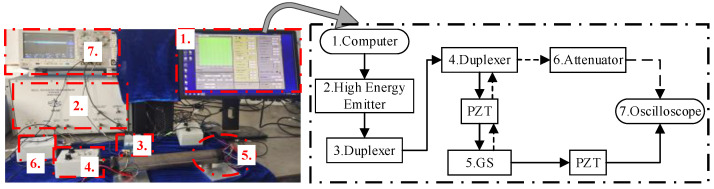
Experimental setup.

Guide waves were excited with the use of the 33220A function generator. An MDO3022 hybrid oscilloscope (from Tektronix, Shanghai, China) was then used to collect the output signals that were averaged 512 times. The sampling frequency was 5 MHz, satisfying the Nyquist—Shannon sampling theorem, and a total length of 100,000 points was collected. A diagram of the experimental setup is given in Figure 8. The PZTs were shear-type, and their cross-section sizes were 4 mm × 8 mm × 10 mm. The eight PZTs at each end for the simultaneous transmission and reception of the ultrasonic waves.

### 4.2. Experimental Results

**Figure 9:**
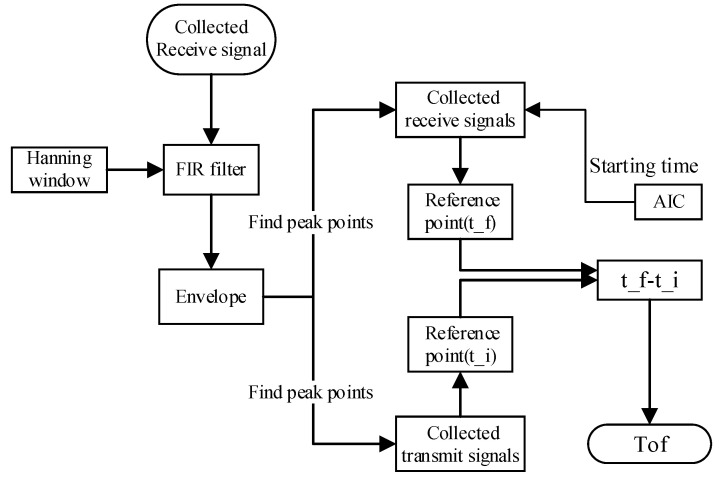
Extraction of the ToF flow chart of the experimental signal.

The detailed process of the ToF for the experimental signal is shown in Figure 9. It can be seen that, first, the signal acquired by the oscilloscope should undergo FIR filtering, followed by using the Hilbert transform to find the extreme points of the received and transmitted signals. The rest of the steps are consistent with the process in Section 3. This is because the signals collected contain noise and DC components, which will significantly reduce the signal quality. The filter used in Figure 9 is the FIR bandpass filter based on the Hanning window function [28]. The passband cut-off frequencies were set to 120 kHz–280 kHz, and the stopband attenuation was 43 dB.

**Figure 10:**
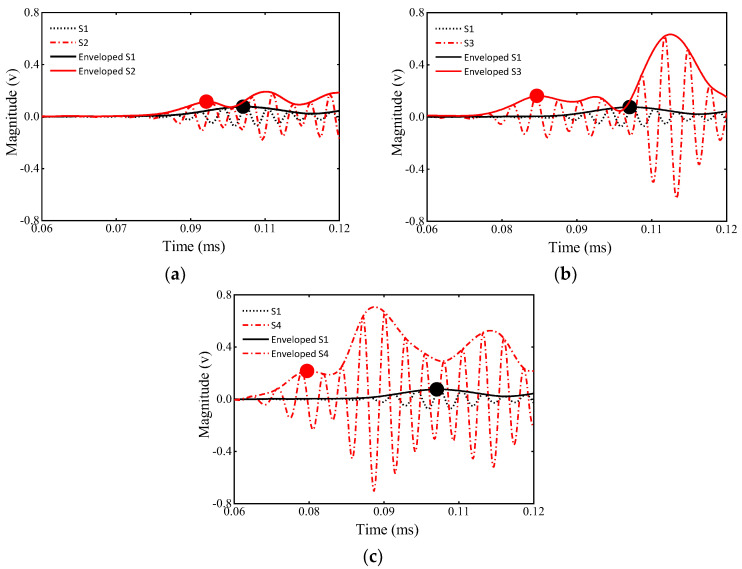
Numerical results of received signals in GSs with the different debonding extents: (**a**) time domain of 0D and 6D signals; (**b**) time domain of 0D and 12D signals; (**c**) time domain of 0D and 18D signals.

The experimental signals filtered by the FIR of four specimens are presented in Figure 10, where the healthy specimen is used as a baseline. The circles represent the peak points of the first wave, which are shifted forward with an increasing defect size. The calculated ToF values are displayed in Table 5. 

**Figure 11:**
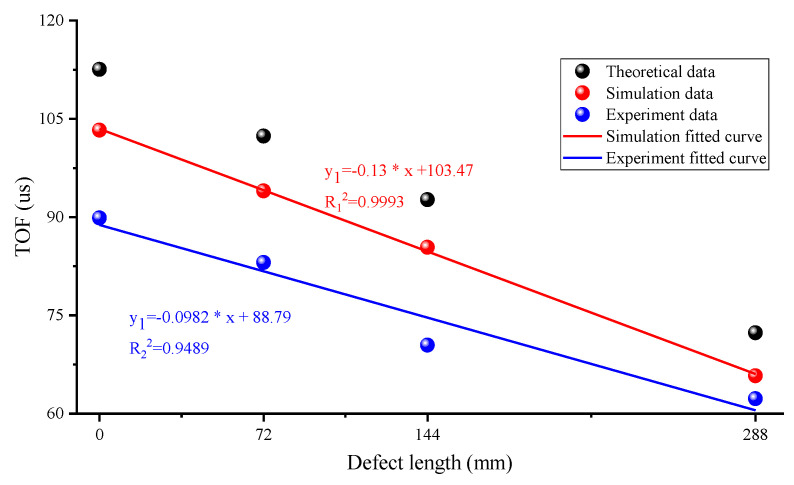
Fitted results between the debonding size and the ToF for simulation analysis and experimental testing.

Figure 11 shows that all the fit variances exceeded 0.95, clearly illustrating a linear relationship between the ToF and the defect sizes. Therefore, it can be concluded that the longitudinal mode is very sensitive to the grout defects occurring in the GS.

### 4.3. Adaptation of Frequency with GS Size

**Figure 12:**
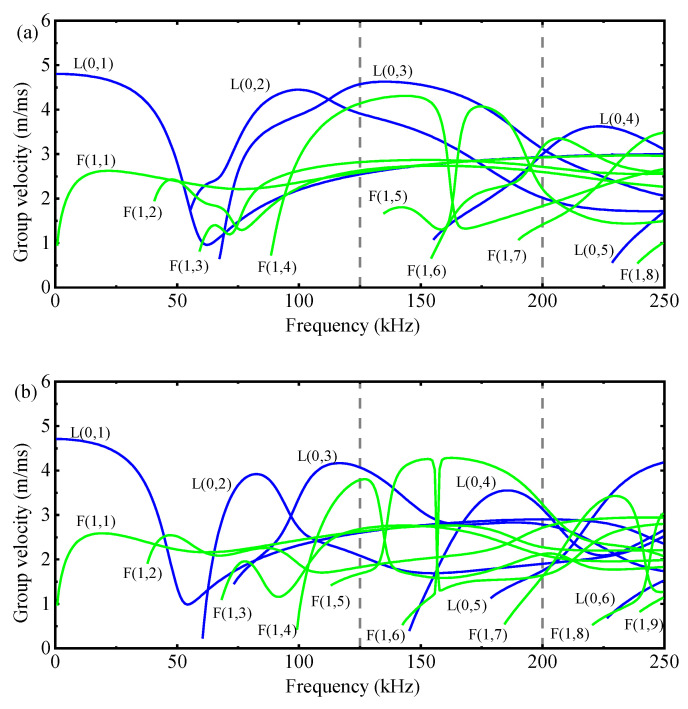

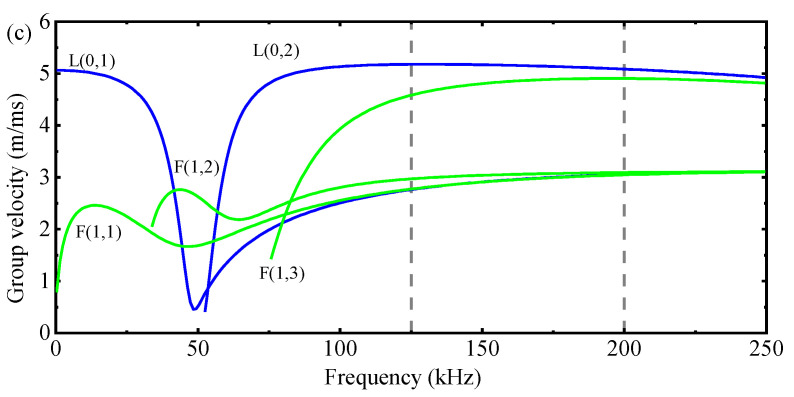
Dispersion curves (group velocity—frequency) for (**a**) Structure I; (**b**) Structure II; and (**c**) Structure III of the GS_12.

In this paper, a GS of one size is only verified by theory, a simulation, and an experiment. In addition, 200 kHz was only verified in the GS of 18 mm (GS_18) and may not be suitable for GSs of other sizes. For example, consider a GS with a rebar diameter of 12 mm (GS_12), a steel casing inner diameter of 28 mm, and a steel casing outer diameter of 38 mm. Figure 12 shows that GS_12, which has a rebar diameter of 12 mm, has fewer modes than GS_18, which has a rebar diameter of 18 mm, due to its smaller size. Furthermore, the velocities of the modes also vary accordingly. Thus, 200 kHz is no longer optimal for debonding detection in GS_12, as the working mode is influenced by the flexural wave mode.

Fortuitously, Section 2.3 provides a frequency selection procedure that accounts for scattering attenuation, the guided wave mode, and resolution. Based on Figure 12, it is evident that 125 kHz serves as the optimal operating frequency for the debonding detection of GS_12. In practical engineering, the rebar size can vary from 10 mm to 40 mm or even larger. Nevertheless, the GS remains a multilayer cylindrical waveguide structure, and its dispersion curves are computed in a similar manner across this size range. Once various dispersion curves are obtained, the frequency selection criteria and debonding detection method introduced in this paper remain applicable. Consequently, the method presented in this paper is adaptable to a wide range of GS sizes. Furthermore, this approach can be extended to other debonding detection applications.

## 5. Conclusions and Future Work

In this paper, the results of theoretical, numerical, and experimental investigations of guided wave propagation in GSs with grout defects in the form of debonding between a steel sleeve and grout are presented. The theoretical wave propagation phenomenon was used to derive the relationships between the ToF of the first wave packet and the defect sizes. Both a simulation and an experiment then confirmed the theoretical relationship. Some main conclusions and contributions are as follows:

1. Grout defecting could be efficiently monitored with the use of PZTs wrapped around steel sleeves without the need to damage the grout and rebar. The defect development led to changes in the wave velocity measured in different specimens;
2. In the ToF extraction process, the Hilbert transform and the AIC algorithm could be used. It is worth noting that the ToF processing of the experimental signals was different from that of the simulated signals due to the addition of a filtering process. The selected Hanning window modulated FIR could effectively eliminate the noise and DC components from the equipment and the environment;
3. The defecting length could be determined based on the measured ToF of the first wave; however, a slight difference in the ToF values was obtained in the theory, simulation, and experiment. Therefore, to accurately evaluate the GS defect, it is recommended to use the ToF determined experimentally on an undamaged object;
4. The working center frequencies for different sizes of GS should be different because the size will affect the dispersion curves. However, the selection principle still needs to consider the three factors proposed in this paper: scattering attenuation, the guided wave mode, and resolution.

It should be noted that the current study is based on a type of GS defect in the lab environment. Issues related to the monitoring of multiple defects in engineering will be investigated in the future.

## Figures and Tables

**Table 1:** Dimensions of the test specimens (unit: mm).

Specimen	L_1_	L_2_	D	d	d_1_	d_2_			L_D_	
S_n	162	16	46	18	3	2	(0	72	144	288)

n is 1, 2, 3, and 4 and represents a defect length (LD) of 0, 72, 144, and 288, respectively. d_1_ represents the thickness of the steel casing, while d_2_ represents the thickness of the defect, and d represents the diameter of the rebar.

**Table 2:** Material properties of the GS in the theoretical study.

Materials Properties	Density (kg/m^3^)	Young’s Modulus (Gpa)	Poisson’s Ratio
Steel sleeve	7850	210	0.3
Steel rebar	7850	210	0.3
Grout	2400	30	0.2

**Table 3:** Theoretical velocities for the three modes at 200 kHz.

Velocity (m/ms)	L (0, 1)	L (0, 2)	L (0, 3)	L (0, 4)
Structure I	2.72	1.78	1.71	3.01
Structure II	2.72	2.09	1.62	3.27
Structure III	2.93	5.23	-	-

**Table 4:** Theoretical results of the Tof values.

Velocity (m/ms)	S1	S2	S3	S4
Defecting length	0	72	144	288
ToF(us)	103.27	93.97	85.37	65.77

**Table 5:** Experimental results of the ToF values.

Velocity (m/ms)	S1	S2	S3	S4
Defecting length	0	72	144	288
ToF(us)	89.87	83.07	70.47	62.27

## Data Availability

The data presented in this study are available on request from the corresponding author.
